# Experiences of living with binge eating disorder and facilitators of recovery processes: a qualitative study

**DOI:** 10.1186/s40337-023-00929-2

**Published:** 2023-11-14

**Authors:** Marit Fjerdingren Bremer, Lisa Garnweidner-Holme, Linda Nesse, Marianne Molin

**Affiliations:** 1 Department of Public Health Science, Faculty of Landscape and Society, Norwegian University of Life Sciences, 1433 Ås, Norway; 2 Department of Nursing and Health Promotion, Faculty of Health Sciences, Oslo Metropolitan University, P.O. Box 4, St. Olavs Plass, 0130 Oslo, Norway; 3 SERAF, Norwegian Centre for Addiction Research, University of Oslo, Oslo, Norway; 4 Department of Health and Exercise, Faculty of Health Sciences, Kristiania University College, Oslo, Norway

**Keywords:** Binge eating disorder, Eating disorders, Qualitative study, Lived experience, First-person perspectives, Recovery

## Abstract

**Background:**

Binge eating disorder (BED) is the most prevalent eating disorder worldwide. BED is often associated with low quality of life and mental health problems. Given the complexity of the disorder, recovery may be challenging. Since BED was only recently specified as a diagnostic category by the World Health Organization (2021), little is known about how patients experience living with BED in everyday life. This study aimed to explore how patients experience living with BED and to investigate factors perceived as facilitating recovery.

**Method:**

Individual interviews were conducted with six patients in a rehabilitation programme for recovery from BED. Interviews were conducted digitally and verbally transcribed between December 2020 and January 2021. The analysis was based on Malterud’s systematic text condensation.

**Results:**

Being diagnosed with BED could be experienced as a relief. The participants perceived living with BED as a challenging addiction. They struggled with a low self-image and experienced a lack of understanding from others, resulting in shame. Self-compassion and social support from friends and family and through participation in a rehabilitation programme were important facilitators of recovery.

**Conclusion:**

Participants perceived living with BED as a challenging addiction. They struggled with low self-esteem and experienced a lack of understanding from others, resulting in shame. Being diagnosed with BED was perceived as a relief. They appreciated that issues related to mental health were addressed during rehabilitation to better understand the complexity of BED. Knowledge about BED, as well as the difficulties of living with BED among family members and friends might help patients with BED feel less ashamed of their disorder and could thus contribute to increased self-compassion.

**Supplementary Information:**

The online version contains supplementary material available at 10.1186/s40337-023-00929-2.

## Abstract (plain language summary)

We interviewed six patients with binge eating disorder (BED) about their experiences living with BED, which is the most prevalent eating disorder worldwide. However, difficulties diagnosing patients with BED and a lack of knowledge about BED among healthcare professionals make it challenging to provide patients with appropriate help to recover from BED. The participants in our study participated in a rehabilitation programme for BED. They experienced living with BED as a challenging addiction. Low self-image and others’ lack of understanding made the individuals ashamed of their eating disorders. Self-compassion and social support through taking part in the rehabilitation programme were important facilitators of recovery. This study indicates that more knowledge about BED among family members, friends and healthcare professionals and social support are notable facilitators for recovering from BED.

**Supplementary Information:**

The online version contains supplementary material available at 10.1186/s40337-023-00929-2.

## Article Highlights

- Even though BED is the most prevalent eating disorder, we have limited knowledge about how patients experience living with BED and their recovery processes
- Patients with BED described the disorder as a challenging addiction
- Low self-image and lack of understanding by others made the participants ashamed of their disordered eating behaviours
- Self-compassion and social support were perceived as core facilitators of recovery

**Supplementary Information:**

The online version contains supplementary material available at 10.1186/s40337-023-00929-2.

## Background

The key characteristics of binge eating disorder (BED) are the tendency to engage in binge eating episodes during which excessive amounts of food are consumed in a short period of time, paired with a subjective sense of loss of control [1]. BED was first recognised as a diagnostic category in the fifth version of the American Diagnostic and Statistical Model of Mental Disorders (DSM) in 2013 [2]. In the European System’s International Classification of Diseases, BED was first specified in 2018 [3]. The lifetime prevalence of BED is estimated to between 1.5 and 1.9%, making it the most prevalent of the eating disorders [4, 5]. Although BED is considered the most common eating disorder, it can be argued to be the eating disorder that receives the least attention in mental health care. Several models of environmental factors contributing to BED have been proposed [6]. These for instance include media exposure, thin-ideal internalisation, and personality traits such as negative emotionality [6]. People with overweight or obesity appear to be at particular risk of developing BED although the directionality in the relationship between overweight, obesity and BED is complex and unclear [7].

Recovery from eating disorders is a non-linear process that includes psychological and social changes, including experiences of empowerment, relationships with others, as well as improvements in body image and reductions in disordered eating patterns [8]. Given the complexity of BED, recovery can be a challenging process [9]. Recovery rates, on average, remain below 50% and largely depend on how recovery is defined [10]. Recovery from BED may be understood and defined differently by patients and health professionals [10].

There is an increasing awareness of BED in the research literature on eating disorders, with several studies exploring patients’ positive and negative experiences of participation in treatment and rehabilitation [11–14]. However, there appears to be fewer studies on patients’ experiences of living with BED in everyday life [15–19]. In qualitative studies, patients have described living with BED as characterized by experiences of guilt and shame, as well as a loss of control [15–19]. However, accepting the disorder and being validated by others have been described as important steps in the recovery process [17]. Furthermore, psychotherapy and person-centred treatment may facilitate recovery [15–17]. Although some studies have investigated patients’ experiences with recovery from BED [8, 17, 20, 21], we have limited in-depth knowledge on facilitators of recovery. Knowledge about how patients experience living with and recovering from BED may be important for better informing our understanding of the influence of BED on everyday life and for tailoring treatment to best promote recovery [22]. This study explores how persons with BED experience living with this eating disorder and investigates factors that were perceived as facilitating recovery.

## Methods

### Design and data collection

Semi-structured individual interviews were conducted by MFB between December 2020 and January 2021. MFB holds a master’s degree in public health science and a bachelor’s degree in public nutrition. MFB currently works at a rehabilitation centre as a nutritionist with patients with obesity. The individuals in this study were recruited from another rehabilitation centre and MFB did not have former knowledge to the participants. Due to the COVID-19 pandemic, the interviews took place online using a digital platform called Visiba Care (visibacare.com), an application or web interface that offers secure communication through video. The interview guide (Additional file 1) was developed by MFB, LN and MM. LN is a clinical psychologist with a PhD in public health science who works in addiction research. MM holds a PhD in nutrition and is a professor in public health and public health nutrition. The themes in the interview guide were developed inductively guided by the research questions of the investigators. The interview guide was pilot tested with a patient with BED. The pilot interview did not change the interview guide. Hence, the pilot interview was included in the sample and analysis of this article. 11 participants attending the rehabilitation programme were invited to participate in the study. 6 agreed to participate. We did not include more participants because we reached information power [23], due to these 6 informants provided very relevant information for the actual research questions in the study. Before participation, the interviewees gave their written informed consent. Recruitment continued until we reached informational power related to the richness of the data [23]. Interviews were audio-recorded with a Dictaphone application [24] and lasted 45–60 min. The interviews were transcribed verbatim by MFB. All the authors read the transcribed interviews. The study was conducted in accordance with COREQ guidelines [25].

### Participants and setting

The participants were all women between 30 and 70 years old. In Norway, persons who have a Body Mass Index (BMI) > 40 without comorbidities or a BMI > 35 with comorbidities qualify for treatment at rehabilitation centres [26]. In some of these centres, patients are screened for eating disorders to identify the potential coexistence of BED. Participants in this study were in treatment for obesity at one of these rehabilitation centres. Based on screening procedures after entering rehabilitation, patients who experienced co-occurring challenges with binge eating were offered participation in a rehabilitation programme focusing on coping with and recovering from BED. The screening process consisted of six questionnaires and a consultation with a psychologist. A clinical assessment was made of whether the person met BED criteria. The questionnaires explored the patients’ eating behaviours and thoughts and feelings related to food. Two questionnaires mapped the patients’ mental health, including anxiety and depressive symptoms.

As part of the rehabilitation programme, sessions were held once a week over three months. The programme involved individual and group-based sessions, with 10 participants, about behaviour change, physical activity, diet, mental health, motivation and empowerment. The group-based sessions were led by a specialist in clinical psychology and a clinical nutritionist. The group-based sessions focused on challenges with binge eating, and important parts of the group discussions were self-esteem, causes and triggers of binging, knowledge of physiological mechanisms, understanding of thoughts and emotions’ influence on behaviours, and further work on recovery. Respondents were given fictive names in the presentation of the results to secure their privacy.

### Analysis

The analysis was conducted by MFB and was guided by Malterud’s systematic text condensation [27], a descriptive and explorative method inspired by phenomenology. LGH, LN and MM assisted with the analysis. The analysis involved the following steps: (1) reading all the transcribed interviews to obtain an overall impression and rereading them with a focus on the study’s aim; (2) identifying and sorting meaning units representing aspects of participants’ lived experiences with BED and perceived facilitators for recovery and coding; (3) condensing the contents and meanings of each coded group and (4) synthesising the contents of each code group to generalise descriptions and concepts. The process to formulate meaning units and the subsequent coding of the content and meaning involved discussion and clearance of the text. The main focus was to discuss understanding of the text, compare main impressions and themes, which again could provide an overview of similarities and differences. We highlighted recurring citations and citations that gave information on equal topics.

## Results

**Fig. 1:**
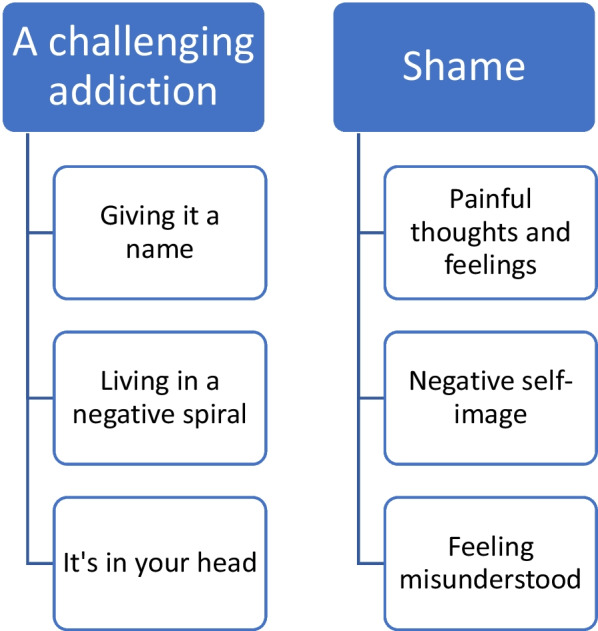
Main themes and subthemes concerning the experience of living with BED

We identified the following two main themes related to patients’ experiences with BED (Fig. 1): (1) A *challenging addiction* with the subthemes *giving it a name*, *living in a negative spiral* and *it’s in your head*; and (2) *shame* with the subthemes *painful thoughts and feelings*, *negative self-image* and *feeling misunderstood*. We found three main themes regarding the perceived facilitators of recovery: (1) *recovery is a long process* with the subthemes *acceptance of the disorder* and *give yourself time*; (2) *coping* with the subthemes *self-compassion* and *strategies to manage the disorder*; (3) *community* with the subthemes group *affiliation* and *social support*.

### Experiences of living with BED

The participants described living with BED as *a challenging addiction*. Berit explained how difficult it was to stop eating: *‘When I eat, I get happy right there and then, but when I think about it, and the dopamine or whatever it is stops working, I feel completely unsuccessful, and then I think that I can just give up. It is over. I just continue to eat. … I can’t do anything right anyway’.*

*Giving it a name* describes participants’ experienced relief of being diagnosed with BED. The participants experienced BED as a complex condition and a challenging disorder that removed their focus from other notable areas of life. They often told stories of repeated feelings of failure in their management of BED. They felt too embarrassed to tell anyone in their lives about their diagnoses and, thus, kept it a secret, even though they thought their family members already probably knew. Their frustration with not being able to control their eating was described as confusing and time consuming. They felt hopeless and stupid. However, being diagnosed with BED was often described as a relief, which Nora expressed:

*‘It’s actually been really nice. (...) I was referred because of my overweight, uhh, and based on mapping and such, I was diagnosed with binge eating disorder. And I was about to say, uhh, that I wasn’t completely surprised. I’ve realised in a way that there has been a problem, uhh, but at the same time, it was kind of good to have it confirmed (...)’.*

All participants were diagnosed with BED at the rehabilitation centre.

The participants described living with BED as *a negative spiral* that was difficult to escape and characterised by periods of guilt when they could not control their eating habits. Tuva explained, *‘Yes, it’s like I don’t use my head. I don’t do what I’m supposed to, ehh, and I don’t enjoy it. I sit and eat with a guilty conscience’.* Conversely, the participants stated that binging gave them good feelings and satisfaction. These binge eating episodes were considered a reward or a strategy to escape stressful experiences in daily life.

Dealing with binge eating was often viewed in the context of how they otherwise felt in life. A negative spiral was also mentioned concerning weight management experiences. Individuals had experiences in which they lost weight but had trouble maintaining weight loss. This led to dissatisfaction and hopelessness and resulted in episodes of increased binge eating. Some participants had lived with BED for a long time and had experienced BED as a permanent part of them.

Participants experienced BED as something that *is in your head*, as Pia expressed: *‘At least it starts there, that the body is a symptom of what’s in the head. I think that about my suffering, that the physical kind of reflects the mental’.* It was vital for participants to understand the connection between physical and mental challenges and how these affect each other. Negative thoughts and feelings often led to binge eating episodes, and subjects appreciated the focus on mental health in the rehabilitation centre to learn strategies to cope and choose differently.

All participants associated BED with *shame*, as illustrated by Berit’s statement:

*‘It is very taboo, very taboo. I try to hide it from everyone. When we’re with others, I don’t eat more than others, but when I’m at home and no one sees me, that’s when I eat. So, it’s tiring, and you always watch out. You never feel well enough, and uhh yeah, it really hurts’.* Shame was often described as *painful feelings* and *negative thoughts*. The participants often felt ashamed when other people asked them, *‘Why can’t you just stop eating?’* This question made them feel ashamed of not being in control of their eating behaviours. In this context, the respondents explained that most binge eating episodes occurred when they were alone to avoid feelings of shame. The participants had many negative thoughts and spent much time ruminating about what others thought about them. Thus, shame often related to subjects’ *negative self-image*, as this comment by Pia illustrated: *‘That’s kind of what the body ideals are today, thin and slim, and if you don’t fit in that category, there’s something wrong with you’.*

Several participants described having a negative self-image and critical thoughts about their bodies and behaviours. They mentioned that they already had negative self-images before developing obesity and being diagnosed with BED. Obesity was considered challenging in terms of physical limitations and mental health struggles. They described feelings of not fitting into the bodily ideals in today’s society, where thinness and health are expected.

Living with shame was also connected to a feeling of *being misunderstood* by family members, friends or even health professionals. Berit stated: *‘I had a doctor who said, “You just have to pull yourself together. You just have to eat right”. I think there are probably a lot of doctors who don’t have knowledge about binge eating’.*

Participants experienced little openness about BED. They expressed that they feel it is more common to talk and hear about anorexia and bulimia. Having a less-known eating disorder makes it harder to be open and honest. Some kept the disorder a secret from family and friends, which again worsened their shame and hopelessness.

### Facilitators of recovery processes

Recovery from BED was often considered *a long process* involving *accepting the disorder* and *giving oneself time*. Participants defined ‘recovery’ as the process of reducing binge eating and enhancing coping. Being healthy did not imply the total absence of binge eating episodes, but having greater control over the occurrence and amount of food consumed during binge eating episodes, as Kari explained: *‘It is about coping with it so that it does not happen so often and regularly, but to accept that it can happen once in a while and that it is normal and that you should not feel that you have failed. Because I think that when it happens once, seldom, that I have succeeded in recovering’.* The participants did not perceive recovery from BED as being healthy, since they often had other diseases that they had to handle, such as diabetes.

They perceived it as important to have strategies to manage recovery, as Pia described: ‘*I think that you have to work on it continuously. But I see a change because I have gotten some tools that I can use in such situations, and I have another mindset now. I feel more relaxed’.*

Managing to cope with recurring binge eating episodes was considered an important facilitator of recovery. Participants associated coping with exerting control over their eating behaviours. Many subjects felt more in control with others but felt they could lose it when they were alone, as Silje explained: *‘It’s kind of like how you compare yourself to others and how they manage to control their eating, uhh, and that's what I want, too’.*

The participants often managed to have control by avoiding access to foods that triggered BED (e.g. sweets). Nora said, *‘I have the knowledge to choose the food that’s right for me, and I need to have it available’*. Furthermore, they related coping to ‘inner factors’ that influence their health and quality of life. For instance, focusing on health aspects was considered more important than focusing on weight. Health aspects were also an important motivation for recovery. Several participants explained that pain due to being overweight, such as knee arthrosis, motivated them to control their BED.

In addition, *self-compassion* was often mentioned as a significant facilitator of recovery. Participants gave themselves credit and bragged about periods without binge eating as positively self-reinforcing, often disrupting their negative spirals. Pia explained, *‘Self-compassion is very important for me, hm, being good with myself, being my own best friend and to think about what is good for me. Like, ‘Are episodes with binge eating good for me? No, they are not. It is better for me to go for a walk or to eat fruit’.* However, the participants said that self-compassion requires awareness and practice. They highlighted getting older, gaining life experience and being more mature and reflective as factors that made it easier to give oneself acceptance.

‘Time outs’ from eating were reported as an important *strategy to manage the disorder*. The patients stated that breaks gave them time to reflect on why they were eating, as Berit explained: *‘It has also helped me to wait for 15 min and to eat what you like. Take a 15-min break to see if I really want to eat. Very often, you actually don’t want to. I may start to eat, but then I am at least more aware of eating.’* Another participant stated that it was important not to be too strict with oneself and not to have overly strict rules, such as ‘yes food’ and ‘no food’, to cope with BED. Good eating routines were another factor that facilitated recovery. Outdoor activities, listening to music, reading books, knitting and talking to oneself often helped interviewees to avoid new BED episodes. They appreciated that the present rehabilitation programme focused on mental health, well-being and personal relationships with food. Learning about BED gave them a better understanding that obesity did not just result from a lack of self-control and willpower.

One of the most significant facilitators for managing recovery was a *community* characterised by *group affiliation* and *social support*. All outlined the importance of the community at the rehabilitation centre, as Pia described: *‘It was very good to meet others in the same situation and to get validation that there are more people in the same situation and that you can talk to them openly about these episodes without being judged’.*

Some participants feared how they would cope with BED once they no longer belonged to a rehabilitation programme. The perceived social support of others in the group gave them safety. Nora explained, *‘It was very good to not feel alone (…) to hear that others have the same problems. This made it easier to share my experiences. Being together with others in the same situation makes me feel safe’.* The subjects learned to share BED-related experiences and feelings in the group. For recovery, they also considered it important to learn to share their feelings with others outside the programme, as Nora said: *‘I have been better about talking about my feelings at home, for example “Now I am alone, and I am sad because you are not here”’.*

## Discussion

The participants in this study perceived living with BED as a challenging addiction. Being diagnosed with BED could be a relief; however, a negative self-image and experiencing a lack of understanding from others made the participants ashamed of their disorder. The participants experienced limited openness about BED and mental disorders in their social surroundings. Even though participants were still living with BED, perceived facilitators of recovery were self-compassion and social support received during rehabilitation.

In a study comparing how obese women with and without BED experienced binge eating [28], the authors found that women experienced BED as a form of addiction. In this context, the participants in our study experienced living with BED as characterised by negative thoughts and feelings. A review of research on emotion regulation in BED found that negative emotions play an important role in the onset and maintenance of binge eating [29]. Likewise, the participants in our study perceived living with BED as a rollercoaster ride of emotions, where the distance between positive and negative feelings was short. Experiences of living with BED as a negative spiral was also described in another study of patients with BED in the US [14].

The participants in our study often experienced living with BED as characterized by the shame of not having control over their eating habits and weight. Negative comments from family members or friends about their eating habits or obesity exacerbated shame. The participants also related shame to feeling misunderstood by family members, friends or even health professionals. This finding corroborates studies that found that patients with BED often felt misunderstood by health professionals [8]. There are indications that health professionals have limited knowledge of BED. A cross-sectional study in the US identified low awareness of and knowledge about BED among health professionals.

Shame of not having control was identified as hindering recovery in other studies [17, 29, 30]. For instance, a qualitative study investigating using online messages in a rehabilitation programme for BED found that self-blame promoted a feedback cycle of binging, which was perceived as barrier for recovery [17]. As mentioned in the background, some studies have investigated patients’ experiences with recovery from BED [8, 17, 20, 21]. Our participants experienced recovery as a long process that mainly concerned coping. Interestingly, recovery did not imply being fully recovered from binge eating episodes but rather control over the disorder. We found that self-compassion and social support within a rehabilitation programme were the most important facilitators for recovery. Self-compassion involves developing an accepting relationship with oneself, particularly in instances of perceived failure, inadequacy and personal suffering [31], while social support constitutes the availability of potential supporters, or structural support, and the perception of support, or functional support [32]. Studies have revealed promising results for compassion-focused therapy for recovery from BED [33, 34]. Social support may play an important role in BED recovery process [32, 35, 36]. An Australian mixed-methods study outlined the social support in a Instagram community as important facilitator for recovery [37]. Similarly, social support was also a notable facilitator of group-based recovery for patients with BED, combining guided physical exercise and dietary therapy in a study from Norway [14]. Our participants outlined that for recovery, they considered it important to learn to share their feelings with others outside the programme.

All of our participants outlined the importance of being part in a rehabilitation programme for recovery from BED. Several studies have investigated participants’ experiences with different rehabilitation programmes for BED [12, 14, 17, 37]. For instance, a qualitative study exploring participants’ experiences of a web-based programme for bulimia and BED found that interventions should be flexible, considering individual preferences [38]. The participants in our study described the value of addressing cognitive behavioural change and mental health and appreciated receiving support from an interprofessional team that collaborated in their recovery process. However, it should be acknowledged that all of the participants were overweight or obese before their diagnosis with BED. Their experiences with previous weight-loss programmes might have influenced their preferences for addressing mental health in rehabilitation. Women with BED in the US have also reported appreciating receiving weight-neutral rehabilitation programmes for BED after experiences of being blamed for their weight and health conditions [11]. Thus, rehabilitation programmes for patients with BED should be tailored towards subjectively relevant themes to facilitate recovery.

### Limitations

This study was conducted in a small sample size, which is usual for qualitative research aiming to investigate participants’ experiences [23]. However, it has to be acknowledged that the findings of this study are primarily applicable to the specific setting of the study and perhaps only transferable to patients in similar situations or rehabilitation programmes. Participants were interviewed a short time after they completed the programme. Hence, their responses might have been influenced by the focus of the content in programme in regard to facilitators for recovery. In addition, interviews were conducted digitally, which might have influenced the openness of the participants [39].

## Conclusion and implications for practice

The participants perceived living with BED as a challenging addiction. They struggled with low self-esteem and experienced a lack of understanding from others, resulting in shame. They appreciated that issues related to mental health were addressed during rehabilitation to better understand the complexity of BED. Knowledge about BED as well as the difficulties of living with BED among family members and friends might help patients with BED feel less ashamed of their disorder and could thus contribute to increased self-compassion.Rehabilitation programmes should address social support in order to promote recovery from BED.

### Supplementary Information

**Additional file 1**. Interview guide.

## Data Availability

The data analysis for this manuscript can be made available upon reasonable request by contacting the corresponding author.

## Abbreviations

BED: Binge eating disorder
WHO: World Health Organization
