# Supplementary material for: Experiences of living with binge eating disorder and facilitators of recovery processes: a qualitative study
Source: J Eat Disord. 2023 Nov 14;11:201. doi: 10.1186/s40337-023-00929-2 (PMC10647123; doi:10.1186/s40337-023-00929-2)
Supplement: Supplementary file 1 — Additional file 1. Interview guide. [file 40337_2023_929_MOESM1_ESM.pdf]

INTERVIEWGUIDE

Norges miljø- og biovitenskapelige universitet

Experiences of living with binge eating disorder and facilitators of recovery processes

Aim:

- **How do patients experience living with a binge eating disorder?**
- **Factors that may promote recovery**

Initially:

- Introduce myself. Casual small talk. Tell about the project and the purpose of the study. Inform about confidentiality and how data is processed anonymously. Inform about audio recordings using the mobile app Nettskjema-diktafon, why this is being done, and deletion of data at the end of the study. Inform about the length of the interview 45-60 minutes. Inform that participation in the interview is voluntary and that any questions that do not wish to be answered can be omitted. He/she can withdraw during the interview if desired.
- Thanks for participating.
- Ask if the informant has questions/expectations.

Background questions:

- Gender and age
- For how many years do you consider challenges with food and binge eating?

Main topics Interview:	Question formulation:
**Living with the eating disorder**	• Can you tell me about how you experience living with the eating disorder?<br>• How do you experience meeting the health service/therapists you have had?<br>• Can you tell us a little about how you experience being met elsewhere in society – as a patient with this eating disorder?<br><br>• How has focusing on your body and weight affected you?<br>• In what way have you experienced feelings related to overeating? (Shame, guilt, and depressed mood?)<br>• Can you talk about specific experiences related to binge episodes
**Factors that may promote recovery**	• What do you perceive as factors that can promote change?<br>• Can you tell us about an example of a day where you cope with the eating disorder.<br>• What motivates you to recovery?<br>• What impact has it had on being in a group with others?<br>• How have you experienced the treatment you have received?<br>• What do you think about dealing with relapse?<br>• How can openness about the problem lead to recovery?

Ending:

Brief summary and explore the participant's experience of the interview. Ensure that the participant feels understood. End with *"Do you have* questions *or other things you'd like to add before we end the interview?"*

- Thank you for your participation
- Provide information that the participant can contact us if there is anything he/she would like to add or have questions that have arisen after the interview.
- Provide information that participants can receive a completed assignment if desired
- Turn off audio recording
